# Serum Galectin-9 Levels Are Associated with Coronary Artery Disease in Chinese Individuals

**DOI:** 10.1155/2015/457167

**Published:** 2015-11-18

**Authors:** Ruirui Zhu, Cheng Liu, Hongxia Tang, Qiutang Zeng, Xiang Wang, Zhengfeng Zhu, Yuzhou Liu, Xiaobo Mao, Yucheng Zhong

**Affiliations:** ^1^Department of Cardiology, Institute of Cardiovascular Disease, Union Hospital, Tongji Medical College, Huazhong University of Science and Technology, Wuhan 430022, China; ^2^Department of Pediatric Infectious and Immunological Diseases, Wuhan Children's Hospital, Wuhan 430016, China

## Abstract

*Background*. Recently, several studies suggest that galectin-9 (Gal-9) might play a pivotal role in the pathogenesis of autoimmune diseases. However, the exact role of Gal-9 in atherosclerosis remains to be elucidated. *Methods*. Serum Gal-9, high-sensitivity C-reactive protein (hs-CRP), interferon- (IFN-) *γ*, interleukin- (IL-) 4, IL-17, and transforming growth factor- (TGF-) *β*1 were measured. The effect of Gal-9 on peripheral blood mononuclear cells (PBMC) was investigated in patients with normal coronary artery (NCA). *Results*. The lowest level of Gal-9 was found in the ST-segment elevation myocardial infarction (STEMI) group, followed by the non-ST-segment elevation ACS (NSTEACS), the NCA, and the stable angina pectoris (SAP) groups, respectively. Additionally, Gal-9 was found to be independently associated with hs-CRP, lipoprotein(a), and creatinine. Notably, Gal-9 was also noted to be an independent predictor of the Gensini score. Moreover, Gal-9 suppressed T-helper 17 (Th17) and expanded regulatory T cells (Tregs), resulting in decreased IL-17 production and increased secretion of TGF-*β*1. *Conclusions*. Serum Gal-9 is associated with not only coronary artery disease (CAD), but also the severity of coronary arteries stenosis. Gal-9 can expand Tregs and suppress Th17 development in activated PBMC, implying that Gal-9 has the potential to dampen the development of atherosclerosis and may be a new therapy for CAD.

## 1. Introduction

Coronary artery disease (CAD) remains the principal cause of mortality and morbidity around the world [1, 2]. Although the mechanisms are still not entirely resolved, previous studies have demonstrated that an imbalance between T-helper 1- (Th1-) mediated and Th2-mediated immune functions contributes to the development of atherosclerosis [3]. Recently, Th17, which are a newly defined subset different from Th1 and Th2, reactive to autoantigens are involved in the pathogenesis of several autoimmune diseases [4, 5]. In addition to Th1, Th2, and Th17 lineage, regulatory T cells (Tregs), a special subset of CD4^+^ T cells, inhibit atherosclerosis development by attenuating activated T cell responses [6, 7]. Inspiringly, previous clinical and experimental studies from our laboratory have found that Th17/Tregs functional imbalance exists during atherogenesis, indicating that the imbalance of Th17/Tregs plays a critical role in CAD [8, 9]. Hence, these findings have showed that CD4^+^ T cell subpopulations play an important role in the initiation and progression of atherosclerosis.

It is well known that CD4^+^ T cells are divided into different subsets depending on the cytokines they produce. Furthermore, the relationship between atherosclerosis and cytokines is also very complicated. Interferon- (IFN-) *γ*, the principal cytokine of Th1 cells, is proinflammatory and exacerbates atherosclerotic disease, while the Th2 cytokine such as interleukin- (IL-) 4 is considered to be mainly atheroprotective and can neutralize Th1 cytokine activity [10–12]. IL-17 acts in vitro and in vivo as a proinflammatory cytokine [13]. Moreover, Hashmi and Zeng have found that IL-17 and IL-17 induced cytokines (IL-6 and IL-8) were significantly increased in ACS patients [14]. Interestingly, Tregs exert antiatherosclerosis partly by secretion of anti-inflammatory cytokine transforming growth factor (TGF-*β*1), which can in turn induce the expression of forkhead box transcription factor P3 (Foxp3) and promote differentiation of Tregs derived from CD4^+^CD25^−^ T cells [15, 16]. Therefore, these findings suggest that cytokines as those mentioned above are also essential for the formation and progression of the atherosclerotic plaque.

The galectin family is characterized by conserved carbohydrate recognition domains (CRD) that can bind glycosylated proteins [17]. Galectins are involved in a wide range of biologic processes such as cell adhesion and migration, proliferation and apoptosis, tumor biology, and regulation of the immune system [18]. Galectin- (Gal-) 9 is a member of the galectin family of carbohydrate-binding proteins comprising two carbohydrate recognition domains connected by a linker peptide. Gal-9 is mainly expressed by eosinophils, endothelial cells, macrophages, DCs, Kupffer cells, vascular endothelial cells, intestinal epithelial cells, and in particular T lymphocytes [19]. Furthermore, Gal-9 is believed to function predominantly as a multifaceted player in adaptive and innate immunity, especially in T cell development and homeostasis [20–23]. Accumulating evidence shows that Gal-9 induces apoptosis in subsets of differentiated T cells, particularly in Th1 and Th17 cells, and stimulates the activity of Tregs [19, 24–28]. Indeed, treatment with recombinant Gal-9 moderated the progression of experimental autoimmune encephalomyelitis (EAE), arthritis, and diabetes in animal models [19, 29–31], by reducing the number of Th1 and Th17 cells and downregulating circulating IFN-*γ* levels.

T cell immunoglobulin mucin- (Tim-) 3 has been identified as a receptor for Gal-9 [19]. Although Gal-9 can function in a Tim-3-independent fashion, the immune-regulatory effects of Gal-9 are largely attributed to Gal-9-Tim-3 pathway [32–35]. More recently, Foks et al. demonstrated that anti-Tim-3-Ab administration promoted atherosclerotic plaque formation, which indicated that Tim-3-Gal-9 pathway may be concerned with the development of atherosclerosis [36]. Thus, it is reasonable to postulate that Gal-9 may be involved in atherosclerosis based CAD.

In addition, several findings strongly support the original experimental observations that Gal-3 plays an important role in the underlying heart failure (HF) processes and that elevation of Gal-3 is associated with disease progression and poor outcome in HF [37–39]. However, no study has examined the relationship between Gal-9 and CAD. Herein, we investigate serum Gal-9 levels in Chinese patients with CAD, and the severity of coronary arteries stenosis was evaluated by Gensini score. Furthermore, IFN-*γ*, IL-4, IL-17, TGF-*β*1, high-sensitivity C-reactive protein (hs-CRP), and the classical atherosclerosis risk factors were evaluated. Additionally, Th1, Th2, Th17, and CD4^+^CD25^+^Foxp3^+^ Tregs differentiation were detected in PBMC cultures exposed to Gal-9.

## 2. Materials and Methods

### 2.1. Study Participants

232 patients presenting at the Department of Cardiology of Huazhong University of Science and Technology Affiliated Union Hospital between Jan. 2013 and Dec. 2013 with suspected or established CAD were recruited, including 149 males and 83 females. They were divided into four groups: (1) the control group, which consisted of 50 subjects with NCA (28 men and 22 women, mean age 56.7 ± 11.7); (2) SAP group (25 men and 15 women, mean age 62.4 ± 10.0, inclusion criteria: typical chest discomfort that was associated with horizontal or downsloping ST-segment depression >1 mm in an exercise test); (3) NSTEACS group (60 men and 30 women, mean age 60.6 ± 9.9, which included unstable angina pectoris (UAP) and non-ST-segment elevation myocardial infarction (NSTEMI), inclusion criteria: chest pain at rest with definite ischemic electrocardiographic changes, T-wave inversions and/or ST-segment changes, or typical ischemic chest pain which persists for more than 20 min, elevated serum myocardial necrosis markers concentration and dynamic evolution, but not the typical ST-elevation electrocardiogram); (4) STEMI group (36 men and 16 women, mean age 54.1 ± 12.3, inclusion criteria: myocardial infarction that was confirmed by a significant increase of serum creatine kinase MB, troponin I levels, and elevation of ST-segment). Furthermore, all participants underwent coronary angiography after admission and completed a standardized questionnaire about previous and present illness, medication history, and smoking status. Cardiovascular-interrelated factors, such as age, body mass index (BMI), gender, and ejection fraction (EF) were estimated via physical examination, ultrasonic cardiography (UCG), and electrocardiogram (ECG), respectively. Patients with the following characteristics were excluded from study enrollment: valvular heart disease, thromboembolism, autoimmune diseases (systemic lupus erythematosus, rheumatoid arthritis, etc.), collagen disease, disseminated intravascular coagulation, severe liver and kidney disease, symptomatic heart failure, trauma or surgery, or malignant disease.

The study was approved by the Ethics Committee of Tongji Medical College of Huazhong University of Science and Technology, and all participants were provided written informed consent prior to study entry.

### 2.2. Diagnostic Criteria

Two experienced cardiologists, who were not aware of the patients' clinical and biochemical results, visually examined angiographic images to assess the extent and severity of CAD. CAD diagnosis was made according to the presence of ≥50% stenosis in ≥1 main coronary artery. The stenosis severity of CAD was assessed by Gensini score as previously described [40].

### 2.3. Biochemical Measurements

In the AMI group, blood samples were immediately obtained after admission to the department. And blood samples of the other patients were acquired on the next morning after admission. The blood was drawn with a 21-gauge needle for clean venipuncture of an antecubital vein and the samples were collected into sodium heparin vacutainers (Becton-Dickinson). After centrifugation, the serum obtained was stored at −80°C until further use to measure the concentration of Gal-9. The level of Gal-9 was measured by ELISA according to the manufacturer's instructions (R&D systems). In addition, the levels of IFN-*γ*, IL-17 (Bolingkewei Biotechnology, China), IL-4, hs-CRP (Huijia Biotechnology, China), and TGF-*β*1 (Fumeng Gene Biotechnology, China) were measured by ELISA, following the manufacturer's instructions. The minimal detectable concentrations were 28 pg/mL for Gal-9, 1.8 pg/mL for IFN-*γ*, 1.8 pg/mL for IL-17, 0.4 pg/mL for IL-4, 22 pg/mL for hs-CRP, and 8 pg/mL for TGF-*β*1. The intra-assay and interassay coefficients of variation for all ELISA were <5% and <10%, respectively. All samples were measured in duplicate.

All the other biochemical measurements, including serum total cholesterol (TC), triglyceride (TG), low-density lipoprotein cholesterol (LDL-C), high-density lipoprotein cholesterol (HDL-C), apolipoprotein A-I (ApoA-I), apolipoprotein B (ApoB), lipoprotein(a), creatinine, fasting plasma glucose (FPG), uric acid, and cardiac troponin I (cTnI), were carried out by the biochemical laboratory of our cardiovascular institute using standard methods.

### 2.4. PBMCs Preparation and Stimulation

Peripheral blood mononuclear cells (PBMCs) from NCA (excluded from hypertension, diabetes, and dyslipidemia) were isolated by Ficoll-Hypaque density gradient centrifugation (Pharmacia LKB Technology, Uppsala, Sweden). Cells from the interphase were collected and washed twice with Dulbecco's phosphate buffered saline (D-PBS). Remaining erythrocytes were removed using lysis buffer (4.14 g NH_4_Cl, 0.5 g KHCO_3_, and 18.6 mg Na_2_EDTA in 500 mL water, pH = 7.4 and sterilized) for 5 min on ice. PBMCs were resuspended in RPMI1640 (Lonza, Verviers, Belgium) supplemented with 2.5% FCS, penicillin (100 U/mL)/streptomycin (100 U/mL), and sodium pyruvate (1 mM, Sigma). PBMCs were stimulated with anti-human CD3 and anti-human CD28 antibodies (both 2 *μ*g/mL, Sanquin) in the presence of recombinant Gal-9 (M) (0.01–0.1 *μ*M, ProSpec-Tany TechnoGene Ltd., Israel) for 24 h.

### 2.5. Flow Cytometry

Cells were allocated into tubes and washed once in phosphate buffered saline (PBS). For Tregs analysis, the cells were incubated with anti-CD4-FITC-hAb and anti-CD25-APC-hAb (BD Pharmingen). After the surface staining, the cells were stained with anti-Foxp3-PE-hAb (BD Pharmingen) after fixation and permeabilization according to the manufacturer's instructions. For analysis of Th1, Th2, and Th17 cells, the cells were stimulated with phorbol myristate acetate (PMA, 20 ng/mL, Alexis Biochemicals, San Diego, CA) and ionomycin (1 *μ*g/mL, Alexis Biochemicals) for 4 h in the presence of 2 *μ*mol/mL monensin (Alexis Biochemicals). The incubator was set at 37°C under a 5% CO_2_ environment. After culture for 4 hours, the cells were collected for staining according to the instructions. Fixation and permeabilization were necessary before staining with anti-IFN-*γ*-PE-, anti-IL-4-PE-, or anti-IL-17-PE- hAb (BD Pharmingen).

### 2.6. Statistical Analysis

Continuous variables are summarized as mean ± standard deviation (SD), and categorical data are presented as percentages. The Shapiro-Wilk test was used to assess the normality of distribution of continuous variables. In order to compare two groups, continuous variables were tested using the independent samples *t*-test for normally distributed data and the Mann-Whitney *U* test for nonnormally distributed data; the chi-square test was used for categorical variables. When three or more groups were compared, one-way ANOVA was used. If significance was found, Newman-Keuls test was performed for post hoc analysis to detect the difference among groups. Spearman's correlation was used to calculate the correlations between two continuous variables. Multiple stepwise regression analysis was used to evaluate the influence of different variables on Gal-9 and to adjust for covariates. Independent factors were sex, age, cTnI, and the metabolic-related variables including BMI, FPG, lipid profiles, and hs-CRP. To determine the independent predictors for the presence and severity of CAD, all the conventional risk factors associated with CAD were tested in multiple stepwise regression analysis. Statistical analysis was carried out using SPSS 17.0 (SPSS Inc., Chicago, IL, USA). *p* value <0.05 was considered statistically significant.

## 3. Results

### 3.1. Baseline Characteristics of the Study Participants (Tables 1 and 2)

The prevalence of smoking and the levels of TG, lipoprotein(a), FPG, creatinine, hs-CRP, and cTnI were significantly higher in patients with CAD compared to patients with NCA group (all *p* < 0.05). However, other biochemical results, including TC, HDL-C, LDL-C, and uric acid, were similar between NCA and CAD patients. Compared with STEMI group, patients in SAP and NSTEACS groups showed markedly higher HDL-C levels and age and lower levels of lipoprotein(a), FPG, hs-CRP, and cTnI (all *p* < 0.01). Compared to patients with SAP, the use of aspirin, *β*-blockers, and statins was rare in patients with ACS (all *p* < 0.05), whereas the levels of lipoprotein(a) and hs-CRP were markedly higher in patients with ACS (all *p* < 0.01). A significant increase of creatinine levels was observed in patients with STEMI compared with NSTEACS group (*p* < 0.05) and an obvious decrease of uric acid levels was found in patients with STEMI compared to SAP group (*p* < 0.01). Unexpectedly, the distribution of hypertension, diabetes mellitus, dyslipidemia, and family history was similar among patients with ACS and SAP.

### 3.2. Serum Gal-9 Levels in the Four Groups

**Figure 1:**
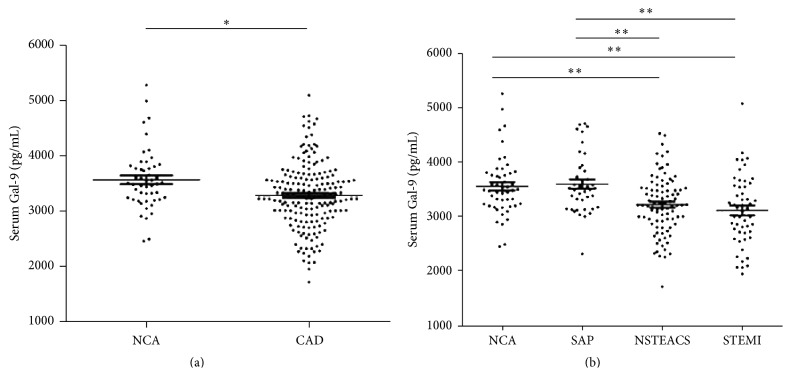
Serum Gal-9 levels in the four groups. (a) Compared with the NCA group, patients with CAD had significantly lower levels of Gal-9 (Figure 1(a)). (b) Serum Gal-9 levels were significantly lower in the STEMI and NSTEACS groups than those in the SAP group or the NCA group (Figure 1(b)). ^*∗*^ *p* < 0.05; ^*∗∗*^ *p* < 0.01.

Among the total 232 study participants, serum Gal-9 levels ranged from 1733.86 to 5259.39 pg/mL. Compared with the NCA group, patients with CAD had significantly lower levels of Gal-9 (3283.55 ± 587.59 versus 3565.97 ± 544.37 pg/mL, *p* < 0.05; Figure 1(a)). In addition, we found that serum Gal-9 levels were significantly lower in the STEMI (3126.36 ± 637.7 pg/mL) and in the NSTEACS groups (3230.21 ± 525.48 pg/mL) than those in the SAP group (3607.91 ± 541.35 pg/mL) or the NCA group (STEMI versus SAP and NSTEACS versus SAP, all *p* < 0.01; STEMI versus NCA and NSTEACS versus NCA, all *p* < 0.01; Figure 1(b)). Interestingly, serum Gal-9 levels did not differ significantly between patients with NSTEACS and STEMI (*p* > 0.05), nor was there a difference between the SAP and NCA groups (*p* > 0.05; Figure 1(b)).

### 3.3. Correlation with Gal-9 and Cytokine Concentrations in the Four Groups

**Figure 2:**
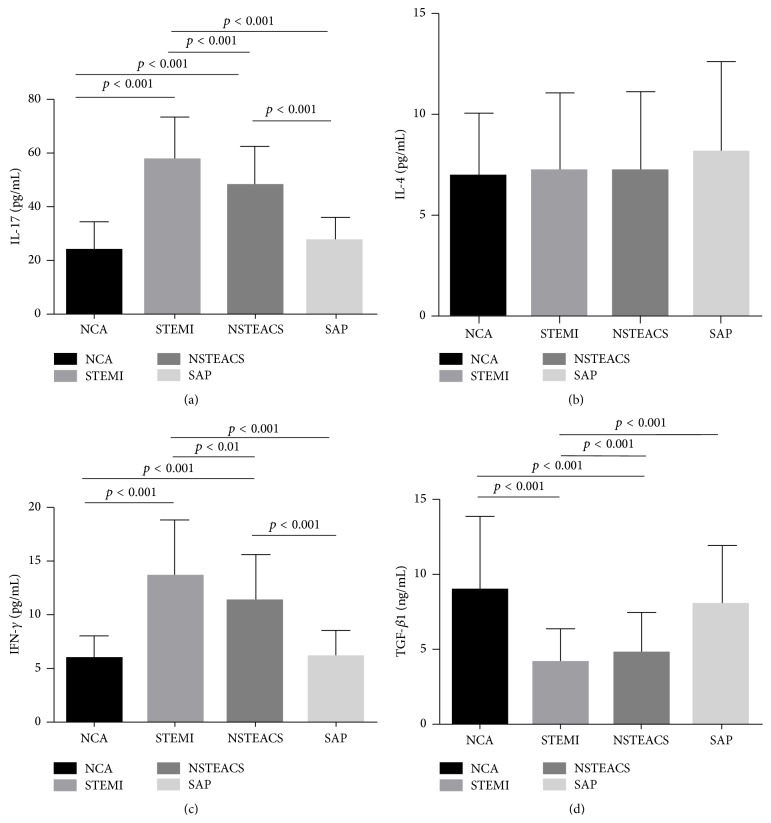
Cytokine concentrations in the four groups. (a) The IL-17 and IFN-*γ* concentrations in patients with STEMI and NSTEACS were significantly higher compared with those in patients with SAP and control (Figures 2(a) and 2(c)). (b) IL-4 concentrations showed no difference in any of the groups (Figure 2(b)). (c) TGF-*β*1 concentrations in patients with STEMI and NSTEACS were significantly lower than those in patients with SAP and control (Figure 2(d)).

**Figure 3:**
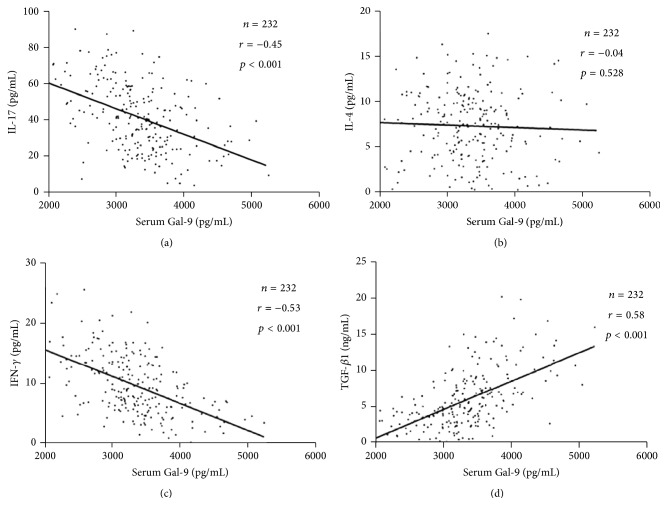
Correlation of Gal-9 levels with cytokines concentrations. (a) Gal-9 levels were shown to be negatively correlated with IL-17 (Figure 3(a)) and IFN-*γ* (Figure 3(c)) but positively associated with TGF-*β*1 (Figure 3(d)). (b) Gal-9 levels do not correlate with IL-4 concentrations (Figure 3(b)).

As shown in Figure 2, serum IL-17, IL-4, IFN-*γ*, and TGF-*β*1 were detected in each group. The IL-17 and IFN-*γ* concentrations in patients with STEMI (IL-17, 57.16 ± 16.14 pg/mL; IFN-*γ*, 13.43 ± 5.29 pg/mL) and NSTEACS (IL-17, 47.68 ± 14.46 pg/mL; IFN-*γ*, 11.28 ± 4.23 pg/mL) were significantly higher compared with those in patients with SAP (IL-17, 26.94 ± 9.09 pg/mL; IFN-*γ*, 6.05 ± 2.41 pg/mL) and NCA groups (IL-17, 24.00 ± 10.14 pg/mL; IFN-*γ*, 5.83 ± 2.12 pg/mL) (all *p* < 0.01; Figures 2(a) and 2(c)). IL-4 concentrations showed no difference in any of the groups (STEMI, 7.25 ± 3.82 pg/mL; NSTEACS, 7.22 ± 3.87 pg/mL; SAP, 8.12 ± 4.48 pg/mL; NCA, 6.83 ± 3.25 pg/mL) (all *p* > 0.05; Figure 2(b)). TGF-*β*1 concentrations in patients with STEMI (4.08 ± 2.13 ng/mL) and NSTEACS (4.73 ± 2.71 ng/mL) were significantly lower than those in patients with SAP (8.03 ± 3.88 ng/mL) and NCA (9.02 ± 4.86 ng/mL) (all *p* < 0.01; Figure 2(d)). Importantly, Gal-9 levels were negatively correlated with IL-17 (*r* = −0.45, *p* < 0.001; Figure 3(a)) and IFN-*γ* (*r* = −0.53, *p* < 0.001; Figure 3(c)) but positively associated with TGF-*β*1 (*r* = 0.58, *p* < 0.001; Figure 3(d)). However, Gal-9 levels showed no correlation with IL-4 concentrations (*r* = −0.04, *p* = 0.528; Figure 3(b)).

### 3.4. The Effects of Gal-9 on PBMCs

**Figure 4:**
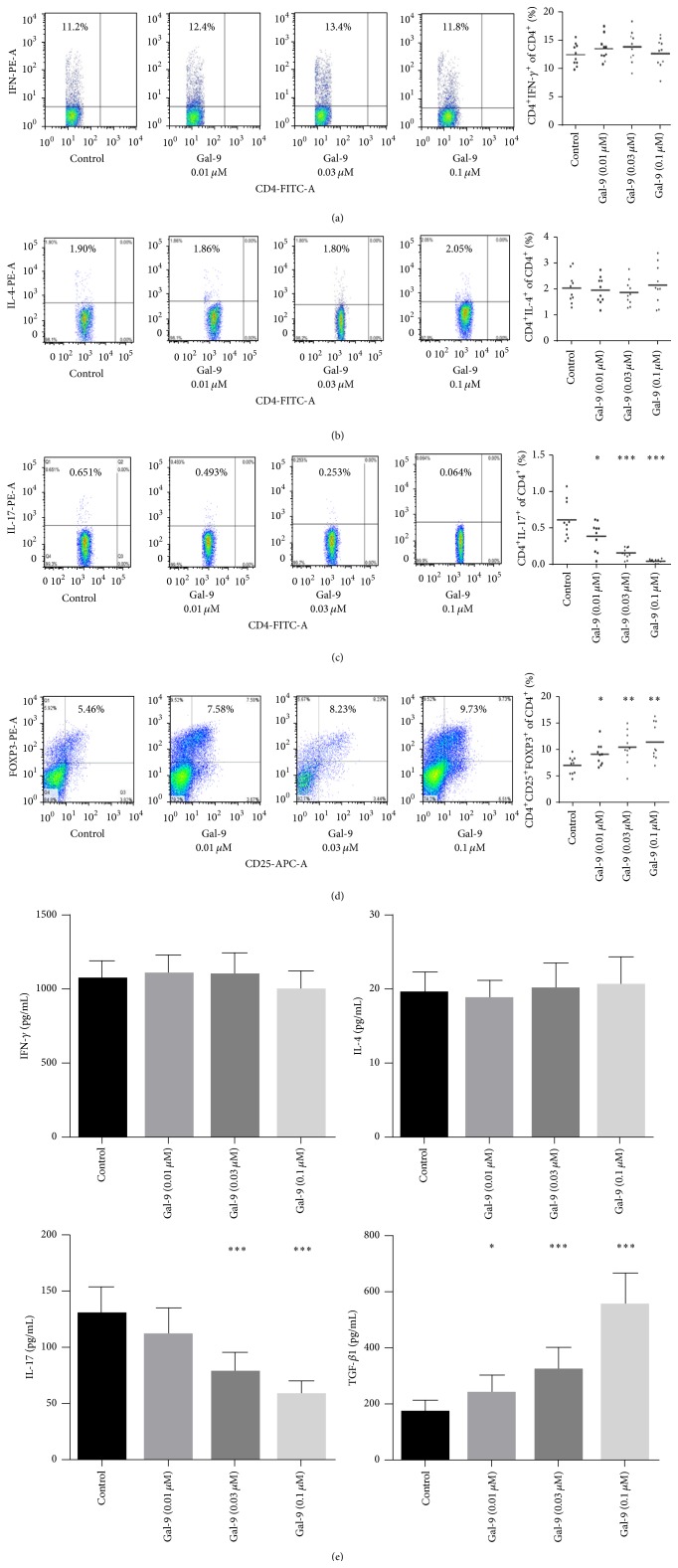
Recombinant Gal-9 enhances the percentage of Tregs and decreases Th17 effector response. CD3/CD28-activated PBMCs were incubated with either medium or increasing concentrations of recombinant Gal-9 for 24 h. The numbers of activated Th1 cells (CD4^+^IFN-*γ* ^+^; (a)), Th2 cells (CD4^+^IL-4^+^; (b)), Th17 cells (CD4^+^IL-17^+^; (c)), and Tregs (CD4^+^CD25^+^Foxp3^+^; (d)) were determined by FACS. Gal-9 increased the development of Tregs and decreases the percentage of Th17 cells dose dependently. (e) Cytokines produced by Gal-9 exposed PBMC were measured in the supernatant by ELISA. Data represent *n* = 10 independent PBMCs of NCA. ^*∗*^ *p* < 0.05, ^*∗∗*^ *p* < 0.01, and ^*∗∗∗*^ *p* < 0.001 compared with control.

To detect the involvement of Gal-9 in the induction of differentiation of human CD4^+^ T cells, CD3/CD28-activated PBMCs were exposed to different concentrations of recombinant Gal-9 (M). Gal-9 expanded the number of CD4^+^CD25^+^Foxp3^+^ Tregs (control, 6.99 ± 1.61%; Gal-9 (0.01 *μ*M), 9.24 ± 2.04%; Gal-9 (0.03 *μ*M), 10.27 ± 3.04%; Gal-9 (0.1 *μ*M), 11.24 ± 3.23%) and decreased the development of Th17 (control, 0.628 ± 0.239%; Gal-9 (0.01 *μ*M), 0.418 ± 0.171%; Gal-9 (0.03 *μ*M), 0.214 ± 0.064%; Gal-9 (0.1 *μ*M), 0.059 ± 0.015%) dose dependently (Figures 4(c) and 4(d)), with resulting increase in secretion of TGF-*β*1 (control, 165.3 ± 48.6 pg/mL; Gal-9 (0.01 *μ*M), 227.8 ± 72.4 pg/mL; Gal-9 (0.03 *μ*M), 312.9 ± 81.7 pg/mL; Gal-9 (0.1 *μ*M), 528.1 ± 97.4 pg/mL) and suppressed IL-17 production (control, 132.4 ± 23.6 pg/mL; Gal-9 (0.01 *μ*M), 110.3 ± 25.7 pg/mL; Gal-9 (0.03 *μ*M), 81.9 ± 18.0 pg/mL; Gal-9 (0.1 *μ*M), 56.6 ± 13.8 pg/mL) (Figure 4(e)). However, the development of Th1 and Th2 cells and secretion of IFN-*γ* and IL-4 in the supernatant were similar in the Gal-9 untreated and treated PBMCs (Figures 4(a), 4(b), and 4(e)).

### 3.5. Correlation of Gal-9 Levels and Anthropometric, Biochemical Variables

**Figure 5:**
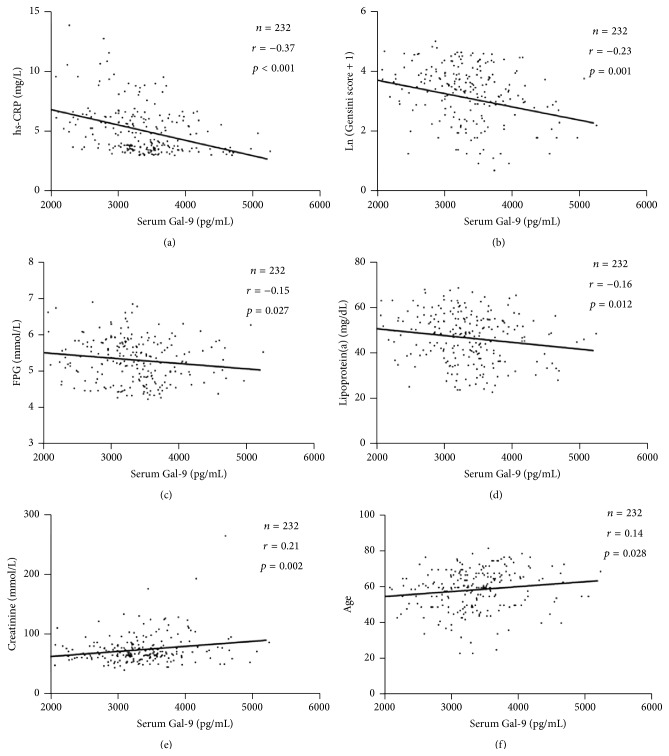
Spearman's correlation of Gal-9 levels with anthropometric and biochemical variables. Gal-9 levels were found to be negatively correlated with hs-CRP (Figure 5(a)), the Gensini score (Figure 5(b)), FPG (Figure 5(c)), and lipoprotein(a) (Figure 5(d)) but positively associated with creatinine (Figure 5(e)) and age (Figure 5(f)).

We carried out a correlation analysis between anthropometric, biochemical variables and Gal-9. As we expected, Gal-9 levels were found to be negatively correlated with hs-CRP (*r* = −0.37, *p* < 0.001; Figure 5(a)), the Gensini score (*r* = −0.23, *p* = 0.001; Figure 5(b)), FPG (*r* = −0.15, *p* = 0.027; Figure 5(c)), and lipoprotein(a) (*r* = −0.16, *p* = 0.012; Figure 5(d)) but positively associated with creatinine (*r* = 0.21, *p* = 0.002; Figure 5(e)) and age (*r* = 0.14, *p* = 0.028; Figure 5(f)).

### 3.6. Multiple Stepwise Regression Analysis

To determine which variables were independently associated with Gal-9, multiple stepwise regression analysis using Gal-9 as the dependent variable identified sex, age, BMI, FPG, TC, TG, HDL-C, LDL-C, hs-CRP, lipoprotein(a), creatinine, uric acid, cTnI, and therapeutic use of statins as independent variables. After adjustments, only hs-CRP (*b* = −72.158, *p* < 0.001), lipoprotein(a) (*b* = 8.333, *p* = 0.045), creatinine (*b* = 5.228, *p* < 0.001), and use of therapeutic statins (*b* = 362.29, *p* < 0.001) retained significance for independently predicting Gal-9 (Table 3). In order to determine which variables were independently associated with Gensini score, multiple stepwise regression analysis was used. Gensini score was the dependent variable, and sex, age, BMI, FPG, lipid profiles, Gal-9, hs-CRP, uric acid, traditional risk factors, and statins were independent variables. After adjustments, only Gal-9 (*b*′ = −0.145, *p* = 0.011), age (*b*′ = 0.276, *p* < 0.001), and lipoprotein(a) (*b*′ = 0.280, *p* < 0.001) were significantly independent predictors of the Gensini score (Table 4).

## 4. Discussion

In the present study, we found that participants with CAD had lower serum Gal-9 levels compared with NCA. Furthermore, serum Gal-9 levels in the ACS group were significantly lower than those in the SAP or NCA group. Specifically, regression analysis showed that Gal-9 was independently associated with the Gensini score. Moreover, Gal-9 was found to be independently associated with hs-CRP, lipoprotein(a), and creatinine. Notably, Gal-9 suppressed T-helper 17 (Th17) and expanded regulatory T cells (Tregs) as analyzed within the activated CD4^+^ T cell population, resulting in decreased IL-17 production and increased secretion of TGF-*β*1. To the best of our knowledge, this is the first study to investigate the relation of serum Gal-9 levels with the presence and the severity of coronary arteries stenosis.

Atherosclerosis is increasingly being considered as a chronic immune-inflammatory state. It has demonstrated that Tregs play an important role in the development of atherosclerosis [41]. Gotsman et al. and Mor et al. have reported that CD4^+^CD25^+^ Tregs deficiency enhanced atherosclerotic lesion development in LDLR^−/−^ mice, and adoptive transfer of CD4^+^CD25^+^ Tregs attenuated the initiation and progression of atherosclerosis in apoE^−/−^ mice [42, 43]. Moreover, our laboratory has detected that Th17/Treg functional imbalance exists in patients with ACS, suggesting a potential role for Th17/Treg imbalance in plaque destabilization and the onset of ACS [8, 9]. Of note, Gal-9 is a multifunctional modulator of T cell immunity with apoptotic effects on Th17 cells and stimulatory activity on Tregs in autoimmune diseases [23]. In line with this publication, we found that Gal-9 was able to induce CD4^+^CD25^+^Foxp3^+^ Tregs development and inhibit Th17 differentiation in activated PBMCs of NCA. Therefore, we speculated that Gal-9 plays an immune-regulatory role in atherosclerosis via its effects on Tregs and Th17 cells.

ACS patients have significantly higher IL-17 and IL-17 induced cytokines (IL-6 and IL-8) [14], while the pleiotropic cytokine IFN-*γ* is a proinflammatory regulator that is expressed at high levels in atherosclerotic lesions [11]. In addition, it has been demonstrated that increased expression of TGF-*β*1 is a stabilizing factor in human atherosclerotic plaques [44]. In this study, we also found that IL-17 and IFN-*γ* concentrations in patients with STEMI and NSTEACS were significantly higher compared with those in patients with SAP and NCA, while TGF-*β*1 concentrations in patients with STEMI and NSTEACS were significantly lower than those in patients with SAP and NCA. These results again confirmed that inflammatory cytokines mentioned above have differential effects in atherosclerosis based CAD. Interestingly, Gal-9 levels were showed to be negatively correlated with IL-17 and IFN-*γ* but positively associated with TGF-*β*1. Furthermore, we found that Gal-9 was able to increase secretion of TGF-*β*1 and decrease IL-17 secretion in activated PBMCs. Consequently, our data in vitro imply a protective role for Gal-9 in atherosclerosis via expanding TGF-*β*1 and suppressing IL-17 secretion. However, the precise effect and mechanism of Gal-9 in atherosclerosis are still to be elucidated in atherosclerosis animal models. Surprisingly, we found that the development of Th1 cells and secretion of IFN-*γ* in the supernatant were similar in Gal-9 untreated and treated PBMCs, since Zhu et al. reported that Gal-9 induces apoptosis in Th1 cells [19]. This discrepancy may be ascribed to the diverse concentrations of Gal-9 and different incubation periods. Additionally, IL-4 concentrations in the supernatant showed no difference in Gal-9 untreated and treated PBMCs. This result further confirms that the effect of IL-4 is still controversial [45, 46], reflecting its dual pro- and anti-inflammatory character. Moreover, there are numerous publications showing that Gal-9 has proinflammatory effects in addition to its immune-suppressive effects, especially through its actions on cells of the innate immune system like DC and NK cells [47, 48]. Thus, these results indicate that Gal-9 has bidirectional effects like many immune-regulatory molecules. But, Gal-9 shows its immune-suppressive characteristics in our study.

Our data showed that CAD patients had lower serum Gal-9 levels than those without CAD, and Gal-9 was independently associated with the Gensini score in patients with CAD. These results suggest that low serum Gal-9 levels are associated with the presence and the severity of coronary arteries stenosis. Previous studies have elucidated that serum biomarkers are recognized as important tools for prediction, diagnosis, risk stratification, and therapeutic decision-marking for patients with CAD [49]. Accumulating studies demonstrated that atherosclerotic lesion instability and rupture induced by inflammation are the major mechanisms of ACS [50, 51]. Recently, a large number of clinical experiments have reported that hs-CRP is not only a biomarker of inflammations and atherosclerosis, but also a marker of atheromatous plaque vulnerability [52–54]. In our study, patients with ACS had higher serum hs-CRP levels compared to patients with SAP. Of note, Gal-9 was found to be independently associated with hs-CRP. Hence, low serum concentrations of Gal-9 may take part in the onset of ACS and may act as a predictor of atheromatous plaque vulnerability. However, whether a causal relationship exists between the two processes is still to be studied. Several lines of evidence indicate that lipoprotein(a) is an independent risk factor for patients with CAD [55, 56]. Interestingly, we found that Gal-9 was independently associated with lipoprotein(a). All these findings together suggest that Gal-9 might be a potential independent biomarker of CAD.

Interestingly, our data showed that serum Gal-9 levels were higher in patients of CAD complicated with T_2_DM than those of CAD without T_2_DM (data not shown). This is in line with Kurose et al.'s study reporting that serum Gal-9 levels are elevated in the patients with type 2 diabetes (T_2_DM) and chronic kidney disease (CKD) [57]. T_2_DM is known to be a risk factor for atherosclerosis and CAD. However, serum Gal-9 levels were decreased in CAD and elevated in the patients with T_2_DM. This paradox could be attributed to the fact that atherosclerosis is highly multifactorial, Tregs and Gal-9 being only some factors among many others. The exact mechanism is still to be elucidated but different diseases may have divergent immune states.

Worth noting is the fact that the serum Gal-9 level seems relatively higher in our study than in the previous studies [22, 57]. The differences among these studies might be explained, at least partly, by different experimental designs, different number and characteristics of participants recruited, ethnical differences, medical treatments, lack of standardization of methods for the determination of serum Gal-9 levels, and different ways in which samples were handled. Nevertheless, several limitations of the present study should be considered. Firstly, the number of study participants (232) was relatively small. Secondly, given the cross-sectional design of the study, the causal relationship between Gal-9 level and the severity of coronary artery stenosis cannot be determined. Thirdly, different methods of assessment of the coronary stenosis and the criterion for diagnosis of the diseased artery may lead to another result. Finally, although we accounted for confounding of traditional cardiovascular risk factors, a potential for uncontrolled or residual confounding that could influence the relationship between Gal-9 and CAD would be plausible.

In conclusion, we have shown that low serum Gal-9 levels are associated with the presence and the severity of coronary arteries stenosis. Importantly, Gal-9 can expand CD4^+^CD25^+^Foxp3^+^ Tregs and inhibit Th17 development in activated PBMCs, leading to increased secretion of TGF-*β*1 and decreased IL-17 secretion. Our observations provide evidence of the role of Gal-9 in CAD and that Gal-9 is an independent negative predictor of ACS, which may also at least partially explain the protection of Gal-9 against coronary atherosclerotic plaque vulnerability. Large-scale population-based clinical studies and further experimental studies are needed to elucidate the exact effect and potential biological mechanisms of Gal-9 in the pathogenesis of CAD and to evaluate whether Gal-9 is a potential novel biomarker of CAD.

## Figures and Tables

**Table 1:** Clinical characteristics of patients.

Characteristics	Control (*n* = 50)	CAD (*n* = 182)	*p*	SAP (*n* = 40)	NSTEACS (*n* = 90)	STEMI (*n* = 52)
Age (years)	56.7 ± 11.7	59.18 ± 11.1	0.178	62.4 ± 10.0	60.6 ± 9.9	54.1 ± 12.3^##&&^
Male/female	28/22	133/49	0.032	25/15	60/30	36/16
BMI	23.6 ± 3.1	24.2 ± 3.5	0.328	23.8 ± 3.1	23.9 ± 3.5	24.9 ± 3.8
Risk factors						
Hypertension, *n* (%)	0	72 (40%)	—	17 (43%)	35 (39%)	20 (39%)
Diabetes, *n* (%)	7 (14%)	24 (13%)	0.882	2 (5%)	13 (14%)	9 (17%)
Dyslipidemia, *n* (%)	0	29 (16%)	—	7 (18%)	16 (18%)	6 (12%)
Smoking, *n* (%)	12 (24%)	119 (65%)	<0.001^*∗∗*^	18 (45%)	61 (68%)^*∗∗*#^	40 (77%)^*∗∗*##^
Family history, *n* (%)	0	39 (21%)	—	7 (18%)	19 (21%)	13 (25%)
Medications						
Aspirin, *n* (%)	0	51 (28%)		15 (38%)	24 (27%)	12 (23%)
Clopidogrel, *n* (%)	0	8 (4%)	—	3 (8%)	2 (2%)	3 (6%)
Beta-blockers, *n* (%)	0	51 (28%)	—	17 (43%)	24 (27%)	10 (19%)^#^
ACEI, *n* (%)	0	53 (29%)	—	10 (25%)	28 (31%)	15 (29%)
ARB, *n* (%)	0	30 (16%)	—	9 (23%)	12 (13%)	9 (17%)
CCB, *n* (%)	0	45 (25%)	—	13 (33%)	20 (22%)	12 (23%)
Statins, *n* (%)	0	46 (25%)	—	18 (45%)	18 (20%)^##^	10 (19%)^##^

^*∗*^ *p* < 0.05 versus control,  ^*∗∗*^ *p* < 0.01 versus control,  ^#^ *p* < 0.05 versus SAP,  ^##^ *p* < 0.01 versus SAP,  ^&^ *p* < 0.05 versus NSTEACS,  ^&&^ *p* < 0.01 versus NSTEACS.

**Table 2:** Biochemical characteristics of patients.

Biochemical characteristics	Control (*n* = 50)	CAD (*n* = 182)	*p*	SAP (*n* = 40)	NSTEACS (*n* = 90)	STEMI (*n* = 52)
TC (mmol/L)	4.45 ± 0.48	4.60 ± 0.57	0.082	4.55 ± 0.55	4.56 ± 0.52	4.72 ± 0.67
TG (mmol/L)	1.42 ± 0.33	1.58 ± 0.34	0.007^*∗∗*^	1.69 ± 0.35^*∗∗*^	1.52 ± 0.33	1.58 ± 0.35
HDL-C (mmol/L)	1.21 ± 0.17	1.17 ± 0.18	0.129	1.26 ± 0.15	1.19 ± 0.18	1.06 ± 0.16^*∗∗*##&&^
LDL-C (mmol/L)	2.39 ± 0.42	2.31 ± 0.58	0.344	2.32 ± 0.40	2.37 ± 0.36	2.19 ± 0.90
Lipoprotein(a) (mg/L)	38.37 ± 10.21	49.30 ± 9.84	<0.001^*∗∗*^	42.59 ± 9.27	48.13 ± 8.87^*∗∗*##^	56.51 ± 7.06^*∗∗*##&&^
FPG (mmol/L)	4.86 ± 0.44	5.41 ± 0.59	<0.001^*∗∗*^	5.28 ± 0.46^*∗∗*^	5.29 ± 0.52^*∗∗*^	5.76 ± 0.66^*∗∗*##&&^
Creatinine (mmol/L)	67.1 ± 16.1	75.8 ± 25.7	0.024^*∗*^	79.1 ± 36.5	70.4 ± 16.7	82.7 ± 27.0^*∗∗*&^
Uric acid (mmol/L)	345.6 ± 38.5	351.8 ± 41.0	0.342	369.1 ± 20.9^*∗*^	350.6 ± 38.5	340.7 ± 51.7^##^
hs-CRP (mg/L)	3.53 ± 0.26	5.54 ± 2.10	<0.001^*∗∗*^	3.42 ± 0.30	5.17 ± 1.01^*∗∗*##^	7.79 ± 2.20^*∗∗*##&&^
cTNI (ng/mL)	0.17 ± 0.06	9.04 ± 17.66	<0.001^*∗∗*^	0.20 ± 0.66	2.38 ± 6.25	27.40 ± 23.56^*∗∗*##&&^

^*∗*^ *p* < 0.05 versus control,  ^*∗∗*^ *p* < 0.01 versus control,  ^#^ *p* < 0.05 versus SAP,  ^##^ *p* < 0.01 versus SAP,  ^&^ *p* < 0.05 versus NSTEACS,  ^&&^ *p* < 0.01 versus NSTEACS.

**Table 3:** Variables independently associated with Gal-9, as identified by multiple regression analysis.

Variable	*b*	SE	*p*
hs-CRP	−72.158	19.623	<0.001
Creatinine	5.228	1.424	<0.001
Lipoprotein(a)	8.333	4.119	0.045
Statin	362.290	98.449	<0.001

Variables included in the original model were sex, age, BMI, FPG, TC, TG, HDL-C, LDL-C, hs-CRP, lipoprotein(a), creatinine, uric acid, cTNI, and therapeutic use of statins.

**Table 4:** Variables independently associated with Gensini score, as identified by multiple regression analysis.

Variable	*b*	SE	*b*′	*p*
Gal-9	−0.285	0.112	−0.145	0.011
Age	0.028	0.006	0.276	<0.001
Lipoprotein(a)	0.030	0.006	0.280	<0.001

The dependent variable was Gensini score [Ln(Gensini score + 1)]. Variables included in the original model were Gal-9, sex, age, BMI, FPG, TC, TG, HDL-C, LDL-C, lipoprotein(a), hs-CRP, cTNI, uric acid, smoking, and therapeutic use of statins.

## Abbreviations

CAD: Coronary artery disease
Gal-9: Galectin-9
ACS: Acute coronary syndrome
NSTEACS: Non-ST-segment elevation ACS
STEMI: ST-segment elevation myocardial infarction
SAP: Stable angina pectoris
NCA: Normal coronary artery
hs-CRP: High-sensitivity C-reactive protein
IFN-*γ*: Interferon-*γ*
IL: Interleukin
TGF-*β*1: Transforming growth factor-*β*1
PBMC: Peripheral blood mononuclear cells
FPG: Fasting plasma glucose
TH: T-helper
Tregs: Regulatory T cells
Tim-3: T cell immunoglobulin mucin-3
HF: Heart failure
BMI: Body mass index
EF: Ejection fraction
UCG: Ultrasonic cardiography
ECG: Electrocardiogram
ELISA: Enzyme-linked immunosorbent assay
TC: Total cholesterol
TG: Triglyceride
LDL-C: Low-density lipoprotein cholesterol
HDL-C: High-density lipoprotein cholesterol
ApoA-I: Apolipoprotein A-I
ApoB: Apolipoprotein B
cTNI: Cardiac troponin I
T_2_DM: Type 2 diabetes
CKD: Chronic kidney disease.
